# Tremor and hand-arm vibration syndrome (HAVS) in road maintenance workers

**DOI:** 10.1007/s00420-016-1175-x

**Published:** 2016-10-28

**Authors:** Rita Bast-Pettersen, Bente Ulvestad, Karl Færden, Thomas Aleksander C. Clemm, Raymond Olsen, Dag Gunnar Ellingsen, Karl-Christian Nordby

**Affiliations:** 1 National Institute of Occupational Health, Oslo, Norway; 2 Department of Environmental and Occupational Medicine, Oslo University Hospital, Oslo, Norway; 3 Mesta AS, Baerum, Norway

**Keywords:** CATSYS Tremor Pen^®^, Hand-arm vibration syndrome (HAVS), Postural tremor, Rest tremor, Cotinine

## Abstract

**Objectives:**

The aim of this study was to evaluate postural and rest tremor among workers using vibrating hand tools, taking into account the possible effects of toxicants such as alcohol and tobacco. A further aim was to study workers diagnosed with hand-arm vibration syndrome (HAVS) at the time of examination.

**Methods:**

This study comprises 103 road maintenance workers, 55 exposed to vibrating hand tools (age 41.0 years; range 21–62) and 48 referents (age 38.5 years; range 19–64). They were examined with the CATSYS Tremor Pen^®^. Exposure to vibrating tools and serum biomarkers of alcohol and tobacco consumption were measured.

**Results:**

Cumulative exposure to vibrating tools was associated with increased postural (*p* < 0.01) and rest tremor (*p* < 0.05) and with a higher Center Frequency of postural tremor (*p* < 0.01) among smokers and users of smokeless tobacco. Rest tremor Center Frequency was higher than postural tremor frequency (*p* < 0.001).

**Conclusions:**

The main findings indicate an association between cumulative exposure to hand-held vibrating tools, tremor parameters and consumption of tobacco products. The hand position is important when testing for tremor. Rest tremor had a higher Center Frequency. Postural tremor was more strongly associated with exposure than rest tremor. The finding of increased tremor among the HAVS subjects indicated that tremor might be a part of the clinical picture of a HAVS diagnosis. As with all cross-sectional studies, inferences should be made with caution when drawing conclusions about associations between exposure and possible effects. Future research using longitudinal design is required to validate the findings of the present study.

## Introduction

Excessive use of hand-held vibrating tools may lead to adverse health effects, including the impairment of hand function. Vibration exposure may lead to hand-arm vibration syndrome (HAVS), which is composed of vascular, neurological and muscular components (Burström et al. 1998; Ye et al. 2015). Typical symptoms include vasospasm of the fingers induced by cold, loss of sensitivity, tingling and paresthesia, and impaired hand function (Heaver et al. 2011). Severe HAVS can lead to difficulties in performing everyday activities (Buhaug et al. 2014), with lowered work ability and quality of life (Gerhardsson and Hagberg 2014; Sauni et al. 2015). HAVS is often diagnosed by clinical examination based on the Stockholm workshop scale (Gemne et al. 1987; Brammer et al. 1987).

Exposure–response relationships between vibration exposure and the vascular (Griffin et al. 2003; Sauni et al. 2009) and neurological (Edlund et al. 2014; Sauni et al. 2009) components of HAVS have been reported. The factors that may affect the risk of developing HAVS symptoms are the tools’ acceleration levels, the duration of exposure, the grip force required, the structure of the work surface, the working posture, the climatic conditions and individual susceptibility (Burström et al. 2006; Griffin 1997; Ye et al. 2015).

Exposure to various neurotoxins has been found to affect tremor due to central nervous system (CNS) effects (Bast-Pettersen et al. 2004; Ellingsen et al. 2006; Lucchini et al. 1999). It has been less studied whether exposure that does not primarily affect the CNS can affect tremor. Because subjects with HAVS have symptoms such as reduced sensory function, tingling and paresthesia, it is of interest whether the disturbance of hand function due to vibration exposure could also lead to increased tremor. However, few studies have examined tremor among vibration-exposed workers. Futatsuka et al. (2005) described tremor among quarry workers in Vietnam, and Bylund et al. (2002) reported tremor among women exposed to hand-arm vibration, but tremor was recorded as subjective complaints without quantitative measures in these studies. In a recent study of 139 male workers exposed to hand-arm vibration in an engineering plant, no changes in quantitatively measured tremor parameters were observed (Edlund et al. 2015). Tobacco consumption was a statistically significant predictor of increased tremor amplitude and, to a certain degree, also a predictor of other tremor parameters in the study, which to our knowledge is the only published study of subjects exposed to hand-arm vibration in which tremor was measured quantitatively.

Tobacco use and alcohol consumption may affect dopaminergic structures of the brain, and previous studies have shown increased tremor among smokers (Bast-Pettersen et al. 2004, 2005; Ellingsen et al. 2006). The use of smokeless tobacco in the form of moist snuff, called “snus,” is also widespread in the Scandinavian countries (Hugoson et al. 2012), but it is difficult to quantify nicotine uptake from these habits through questionnaires.

Nicotine is distributed to most tissues after absorption and also to the brain. Nicotine is mainly metabolized in the liver, and its metabolite cotinine, with a half-life of approximately 16 h (Davis et al. 2015), is regarded as the best predictor of total nicotine intake (Davis et al. 2015; Hukkanen et al. 2005). The plasma concentration of serum carbohydrate-deficient transferrin (sCDT) is increasingly being used as a biomarker of alcohol consumption. This biomarker has a half-life in the blood of approximately 14 days (Tomberg 2010), and a chronic daily intake of 60–80 g of ethanol is considered to increase sCDT above the “normal” level of 1.7% (Bortolotti et al. 2006).

## Tremor

Tremor can be defined as “a rhythmical, involuntary oscillatory movement of a body part” (Findley 1996). A fine, low-amplitude tremor accompanies any voluntary activation of muscle, but under normal circumstances, this physiological tremor can only be detected by sensitive recording devices (Findley 1996). The most important features of tremor are amplitude and frequency together with the activation condition. Physiological hand tremor is suggested to contain two distinct rhythmic components, a mechanical component that depends on the part of the body from which it is recorded (mechanical reflex) and a neurogenic component (Elble 1996; Elble and Koller 1990; Deuschl et al. 2001). The frequency of the mechanical component is largely determined by the inertia and stiffness of the body part, and therefore, the tremor frequency is different for different body parts (Elble 1996). For instance, hand tremor has a frequency of 6–12 Hz (Deuschl et al. 2001; Elble and Koller 1990; Findley 1996), but finger tremor has a frequency of approximately 25 Hz (Deuschl et al. 2001), or 17–30 Hz (Elble 1996). The neurogenic, central oscillation has a frequency of 8–12 Hz and is independent of body mechanics (Elble and Koller 1990; Deuschl et al. 2001).

The International Tremor Foundation has proposed a classification of tremor based on observational tremor classifications and activation conditions (Findley 1996; Deuschl et al. 1998). According to this classification, *rest tremor* occurs when muscles are not voluntarily activated and the body part is completely supported against gravity. In contrast, *action tremor* occurs with voluntary contraction of muscle. Action tremor can be divided into *postural tremor* (present while voluntarily maintaining a position against gravity), *kinetic tremor* (including “intention tremor,” the exacerbation of kinetic tremor toward the end of goal-directed movement and “task-specific kinetic tremor,” for example, writing tremor) and *isometric tremor*.

### Objectives

The objectives of this study were to evaluate postural and rest tremor among workers exposed to hand-held vibrating tools, taking into account the possible effect from lifestyle habits such as alcohol and tobacco consumption. A further aim was to study tremor parameters in workers who were diagnosed with HAVS at the time of the examination and to investigate factors that might increase the likelihood of being diagnosed with HAVS.

## Subjects and methods

The participants in this cross-sectional study were recruited from Norway’s largest road maintenance company. They were invited to participate in a study of health effects related to hand-arm vibration exposure in connection with a regular medical surveillance by the occupational health service. The present study represented an extended examination in addition to their regular health control. One hundred and six workers employed in this nationwide company were invited. All subjects from the rock slope stabilization department (*N* = 26) and all subjects from the guardrail department (*N* = 34) were invited. Two subjects from the rock slope department decided not to participate, and one subject from the guardrail department was excluded due to concurrent illness on the occasion of inclusion. Two subjects employed in the guardrail department had not been exposed to vibration from impact wrenches and were therefore allocated to the reference group. Blue-collar workers from the same company, including two subjects employed in the guardrail department (*N* = 48) were recruited as referents, and all of them accepted to participate.

Thus, the study group consisted of 103 subjects. The participants were examined at the occupational health clinics of the respective facilities between November 25, 2013, and March 3, 2014. Before being examined, the subjects went through a structured telephone-based interview focusing on occupational history, smoking habits, and current and past illnesses. All participants were unexposed at the day of examination.

Participation in the study was voluntary, and informed written consent was obtained from each participant. The study was approved by the Norwegian Regional Ethical Committee for Medical Research (REC South East).

### Work tasks

The rock slope stabilization crew (*N* = 24) perform work tasks such as securing unstable rock faces with rock bolts, mesh and nets and setting up fences as protection from rocks and landslides, using manually operated pneumatic rock drills.

The guardrail crew (*N* = 31) assembles guardrails along roads. They use remote-controlled mobile drilling rigs to drill holes for the poles. The guardrails are manually lifted in place and attached to the poles using hand-held power wrenches.

The referents (*N* = 48) have different work tasks such as filling in holes in roads, assembling road signs, repairing damaged guardrails, cutting vegetation along roads, plowing snow, and cleaning and inspecting roads. Much of the time during a typical working day is spent driving a car or truck. Hand-held vibrating tools are used occasionally but not on a regular basis. The referents were unexposed to rock drills. Some of them may have used impact wrenches in other settings than as guardrail crew, but only to a limited extent.

### Exposure assessment

In order to validate the r.m.s. levels of acceleration according to the values in the “Hand-arm vibration guide to good practice” given as 17 ms^−2^ r.m.s. for rock drills and 7 ms^−2^ r.m.s. for impact wrenches (Griffin et al. 2006), 44 field measurements of the acceleration levels of rock drills and impact wrenches were performed within the present study.

The vibration exposure level during rock drill operation varies with several factors. Among these are the need to use a fixed hand grip to control and press the device toward the drill hole, the angle with the gravity line at which the drill operates, the use of a jack leg, hand jack or iron weight to generate force toward the drill hole, and the hardness of the rock (Table 1).

**Table 1:** Background and exposure data for the 103 workers included in the study

	Exposed (*N* = 55)			Referents (*N* = 48)		
	Arithmetic mean	SD	Min–max	Arithmetic mean	SD	Min–max
Age	41.0	10.6	21–62	38.5	14.5	19–64
Cumulative exposure dose (ms^−2^ r.m.s. x h) Log_10_	3.31	0.78	–	0	0	–
Cumulative exposure dose (ms^−2^ r.m.s. x h)	8340	19,190	4–99,190	0	0	0–0
Height (cm)	180	6	166–193	181	6	170–200
Weight (kg)	91.1	15.1	63–132	91.9	16.5	65–135
Body mass index (kg.m^−2^)	28.0	4.0	20.2–38.6	27.9	4.2	19.0–40.1
Right-handers (%)	89	–	–	90	–	–
Prevalence smoker/user of smokeless tobacco (%)	65	–	–	48	–	–
sNicotine (µg L^−1^)^a^	21	30	0–110	11	21	0–65
sCotinine (µg L^−1^)^a^	426	447	0–1744	235	350	0–1390
sCaffeine (µg L^−1^)^a^	3610	2630	0–12,770	2030	1910	10–6550
Log_10_ sCDT^b^ (%)^a^	−0.17	0.12	–	−0.20	0.12	–
sCDT^b^ (%)^a^	0.70^c^	0.20	0.4–1.4	0.65^d^	0.21	0.4–1.7
HbA1c (%)^a^	5.33	0.27	4.5–5.9	5.22	0.38	4.4–6.6

^a^ *N* = 95; 52 exposed and 43 referents

Measurements of representative acceleration levels during the operation of rock drills across the variable conditions mentioned above were performed according to ISO 5349-2, using recording accelerometer equipment with adaptors interposed between each hand of the operator and the tool, within the glove. Measurements on different rock drill equipment were taken, and average exposure time was estimated based on observations and interviews with the workers. This was done in order to record the actual contact time and integrate the acceleration level that was transferred through the adaptor to the hand during operation (Table 2).

**Table 2:** Partial and total exposure in rock drilling, with different rock drill equipment

Equipment	Average vibration exposure (RMS) ms^−2^	Average daily exposure time (min.)	Number of measurements
Rock drill hand jack	16	20	8
Rock drill jack leg	16	15	12
Standard rock drill with iron weight or vibration dampening in hand grips	14	6	10
Standard rock drill without vibration dampening or iron weight	33	3	7
Other tools	10 (estimated)	1	–
Total: the partial exposures equals an average RMS of 17 ms^−2^	17	45	37

The measurements were taken with two different triaxial accelerometers. The SEN021F accelerometer with a T-adaptor fixed between fingers 2 and 3 connected to a Larson Davis HVM 100 logging vibration level meter with Blaze software (Larson Davis, Depew, NY 140,243, USA) was used for this purpose. Further the accelerometer SV105A with a palm adaptor connected to a logging vibration level meter Svantek SV106 with Swan software (Svantek, 04-0872 Warszawa, Poland) was used. The r.m.s. values of acceleration were computed by integrating software in the measurement equipment, using the area under the curve of frequency-weighted acceleration levels during the time of contact with the tool as the variable of interest.

For the two main exposure categories, the rock drill and the impact wrench, a cumulative dose of acceleration level times lifetime exposure time for each individual was estimated, using the formula Σ*a*_*wi*_·*t*_*i*_ where *a*_*wi*_ = weighted acceleration for each tool (i) in ms^−2^ r.m.s. and *t*_*i*_ = the lifetime exposure to vibration for each tool in hours (*h*) (Bovenzi et al. 1995; Edlund et al. 2014).

The number of workdays was set to 180 per year according to the company’s work schedule. The cumulative exposure was used for exposure in models of associations with outcomes. Information about work with other handheld vibrating tools in present and past employments and in leisure time was also obtained from questionnaires. For these tools, we could not assess the actual exposure which was represented by self-reported data only. The use of other tools was therefore represented by self-reported number of cumulated hours in exposed work (without vibration level) in the models. A dummy variable, based on the use of other hand-held vibrating tools >100 h (yes/no) was made, and this variable was used as an indicator variable in the modeling of exposure-outcome associations.

### Clinical examinations

#### Tremor test

The CATSYS Tremor Pen^®^ (version 7.0, Danish Product Development 2000) was used to measure hand tremor. The test equipment consists of a biaxial micro-accelerometer that is embedded in a low-mass stylus (12 cm × 0.8 cm) and connected to a data logger. Tremor is recorded in a frequency band ranging from 0.9 to 15 Hz. The combined signal from the two perpendicular accelerometers is transformed by the system’s software using Fast Fourier Transformation. Four measures calculated by the CATSYS software were used: Tremor Intensity, Center Frequency, Frequency Dispersion and Harmonic Index.

1. Tremor Intensity (ms^−2^) is defined as the r.m.s of accelerations. The acceleration power spectrum consists of 116 frequency bands approximately 0.12 Hz wide within a range of 0.9–15 Hz. Tremor Intensity is a measure of the magnitude of the tremor as a function of frequency.
2. Center Frequency (Hz): 50% of the power lies above and 50% lies below this frequency (Hz), within the bandwidth of the instrument.
3. Frequency Dispersion (Hz): 68% of the power is dissipated within the Center Frequency ± the upper and lower bounds of the Frequency Dispersion (Hz).
4. Harmonic Index: This index compares the tremor frequency pattern with the pattern of a single harmonic oscillation, which has a Harmonic Index of 1.00. The Harmonic Index decreases when the tremor is composed of many oscillations (Danish Product Development 2000; Bast-Pettersen and Ellingsen 2005).

The recording time is set to 8.2 s as the default (Danish Product Development 2000). However, in the present study, the testing time was set to 16.4, 2 s to stabilize and 14.4 s for recording.

Postural tremor was examined while the subject was sitting in a chair without an armrest and was required to hold the tremor pen as one would hold an ordinary pen in front of the navel with the elbow bent at an angle of 90° and free of any contact with the body or other support. Then, the subject was tested for rest tremor with the tremor pen taped to the hand in the same position as one would hold an ordinary pen. The arm was resting on the table, and the subject was instructed to relax as much as possible during the test session.

### HAVS diagnosis

The subjects were examined using international consensus criteria (Brammer and Lundström 1995; Olsen and Hagberg^1995^; Griffin and Bovenzi 2007; Negro et al. 2008). A vascular HAVS diagnosis was based on a history of finger whiteness reported by the patient and a clinical examination with standardized color chart and finger mapping of vibration-induced white fingers. A neuro-sensorial HAVS diagnosis was based on a neurological examination, including finger mapping of numbness and tingling, vibration perception thresholds on the index and little fingers, and examination of hand function based on a questionnaire. The questionnaire included questions about easily loosing objects, difficulty in fastening of buttons, opening a tight jar lid, picking up coins from a flat surface, pouring liquid from a jug, turning a door knob lever, and putting on a jacket or pullover. As a supplement, a test for manual dexterity (Grooved Pegboard) was applied. The cut-off limit for reduced dexterity measured with the Pegboard test was set to 1 SD below mean (Grooved Pegboard Instruction Manual, Lafayette Instrument Company). Other potential causes for a vascular or neurological diagnosis, e.g., primary Raynaud syndrome, nerve entrapment, injuries or musculoskeletal diseases, were also considered. At the time of the clinical examination, the clinician made a temporary assessment of exposure. The diagnosis was mainly based on the clinical picture of HAVS, as described above.

### Collecting biological samples and determining biomarkers in the serum

Biological samples were collected on the day of the neurobehavioral examinations. Whole blood was collected from the cubital vein with 8 mL Vacutainer tubes without additives (BD Vacutainer, Belliver Industrial Estate, Plymouth, UK). The serum was separated by centrifugation at 2000 g for 10 min. Four samples were pipetted into 4.0 mL NUNC^®^ polypropylene cryotubes (Sigma-Aldrich, St. Louis, Missouri, US) and frozen for storage at the National Institute of Occupational Health, Oslo, Norway at −80 °C until analysis. Due to time pressure and capacity problems on the day of examination, blood samples were available for only 95 subjects.

Carbohydrate-deficient transferrin in the serum was measured at Fürst Medical Laboratory (Oslo, Norway) by capillary electrophoresis using CapillarysTM (Sebia Inc., Georgia, USA). The method’s limit of detection (DL) was 0.4%. A level of <1.7% is considered normal by the laboratory (Ellingsen et al. 2014). Samples for the glycated hemoglobin (HbA1c) measurement were collected in EDTA tubes, and these samples were also analyzed at Fürst Medical Laboratory (Oslo, Norway). A level of 4.0–<6.1% is considered normal by the laboratory.

The sample preparation of cotinine, caffeine and nicotine in serum has previously been described (Ellingsen et al. 2006). However, in this study, two internal standards were used rather than one. To 0.5 mL serum aliquots, we added 100 µl 0.0025 mg mL^−1^ internal standard solution, containing caffeine-^13^C_3_ and cotinine-(methyl-d_3_). A Waters CapLC System (Milford, MA, USA) was used to separate the analytes. A Quattro LC tandem quadrupole MS with positive electrospray ionization (ESI, Micromass, Manchester, UK) was used to separate the analytes and mass spectrometric (MS) detection. Nicotine, cotinine, cotinine-(methyl-d_3_), caffeine and caffeine-^13^C_3_ were monitored as product ions of their respective [M+H]^+^ molecular ions with m/z transitions of 163–132, 177–80, 180–80, 195–138 and 198–140, respectively. The mass spectrometric settings have been described (Ellingsen et al. 2006).

Cotinine, caffeine and nicotine were quantified by adding internal standards and relative comparisons to spiked serum blank samples that were prepared identically. The methods were evaluated over the concentration ranges of 30–20,000 µg nicotine L^−1^ serum, 7.5–15,000 µg caffeine L^−1^ serum and 1.5 to 1000 µg cotinine L^−1^ serum, displaying a coefficient of correlation >0.996. The within-assay (*n* = 6) and between-assay (*n* = 6) precision for nicotine, caffeine and cotinine were <27, <36 and <11%, respectively.

The DL for nicotine was 31 µg nicotine L^−1^ serum; for caffeine, it was 2.1 µg caffeine L^−1^ serum; and for cotinine, it was 1.9 µg cotinine L^−1^ serum. The DL was defined as 2 × standard deviation of the blank.

### Statistics

Continuous variables with a skewed distribution (skewness >2) were log_10_-transformed. The log-transformed values were used in the statistical analysis, and the arithmetic means with standard deviations (SD) for these variables (cumulative dose of exposure, Tremor Intensity of postural tremor for both hands and sCDT) are also presented. Cumulative exposure was log-transformed and treated as a continuous variable. In order to log-transform the values that are equal to zero exposure, their exposure values were set to 1 ms^−2^ r.m.s. h.

Exposure to vibrating tools other than rock drills and impact wrenches was treated as dummy variables in the analysis to assess whether these exposure indicators, even though they were not precisely classified using quantified exposure time, were associated with tremor or, alternatively, confounded the associations between exposure and tremor. A covariate was not regarded as a confounder if the influence on the association between exposure and possible effect was <10%.

Multiple regression analysis was used to assess the age-adjusted associations between cumulative exposure and tremor parameters, all subjects being stratified into users and nonusers of tobacco products, based on the individual concentrations of cotinine in serum (≥5 µg L^−1^). The effects are shown as beta-coefficients (Table 3). A paired *t* test was used to compare postural tremor variables with rest tremor variables. ANOVA was used to compare the subjects who were diagnosed with HAVS with those who were not. Three subjects unexposed to the main tools, and with uncertain information about the underlying exposure, but with a clinical picture resembling HAVS, were not included in the analysis of exposure-outcome associations with HAVS. Logistic regression was used to analyze association between exposure and a diagnosis of HAVS, adjusted for covariates.

**Table 3:** Associations between tremor parameters and cumulative exposure by level of sCotinine

Tremor parameters	sCotinine ≥ 5 (µg L^−1^)^a^ (*N* = 56)				sCotinine < 5 (µg L^−1^)^b^ (*N* = 39)			
	β-Estimate	95% CI			β-Estimate	95% CI		*P*
		Lower bound	Upper bound	*p*		Lower bound	Upper bound	
*Postural tremor dominant hand*								
Tremor Intensity (ms^−2^)	0.019	0.005	0.033	0.009	−0.008	−0.018	0.002	0.12
Center Frequency (Hz)	0.29	0.09	0.50	0.006	0.13	−0.17	0.44	0.37
Frequency Dispersion (Hz)	−0.04	−0.15	0.06	0.42	0.10	−0.08	0.27	0.27
Harmonic Index	−0.004	−0.01	0.003	0.23	−0.001	−0.008	0.006	0.76
*Postural tremor non*-*dominant hand*								
Tremor Intensity (ms^−2^)	0.02	0.007	0.04	0.006	−0.001	−0.008	0.006	0.79
Center Frequency (Hz)	0.40	0.20	0.61	<0.001	0.18	−0.04	0.41	0.10
Frequency Dispersion (Hz)	0.04	−0.06	0.15	0.41	0.05	−0.10	0.20	0.54
Harmonic Index	−0.003	−0.011	0.005	0.49	−0.001	−0.009	0.007	0.81
*Rest tremor dominant hand*								
Tremor Intensity (ms^−2^)	0.013	0.002	0.023	0.02	−0.001	−0.01	0.007	0.73
Center Frequency (Hz)	−0.05	−0.24	0.14	0.60	0.11	−0.09	0.30	0.28
Frequency Dispersion (Hz)	−0.13	−0.27	0.006	0.06	−0.01	−0.20	0.17	0.88
Harmonic Index	0.00009	−0.005	0.005	0.97	−0.003	−0.009	0.003	0.35
*Rest tremor non*-*dominant hand*								
Tremor Intensity (ms^−2^)	0.012	0.003	0.02	0.01	−0.001	−0.01	0.007	0.73
Center Frequency (Hz)	0.09	−0.12	0.30	0.39	0.03	−0.27	0.33	0.85
Frequency Dispersion (Hz)	−0.20	−0.35	−0.05	0.01	0.02	−0.17	0.20	0.84
Harmonic Index	0.002	−0.002	0.007	0.31	0.0	−0.007	0.007	0.97

^a^ sCotinine concentration: 576 µg L^−1^ (range 8.5–1744 µg L^−1^)

^b^ sCotinine concentration: 0 µg L^−1^

The statistical analyses were performed with IBM SPSS^®^, version 22.0, (IBM Corporation, New York). The level of significance was set at *p* ≤ 0.05.

## Results

Table 1 shows the background and exposure data and the biomarker concentrations for the exposed workers and the referents. The groups were similar regarding age, BMI and handedness, but the subjects in the exposed group had substantially higher serum concentrations of cotinine, nicotine and caffeine.

Exposure levels during the operation of rock drills were estimated from typical exposure situations (field measurements) to 17 ms^−2^ root-mean-square (r.m.s.) (Table 2). The levels varied considerably with the conditions of operation. Seven measurements of the acceleration level during use of the impact wrenches were taken. The r.m.s. acceleration levels of hand-arm vibration exposure during this operation were calculated to be 7 ms^−2^ r.m.s. (range 5.4–7.8).

The average time of exposure to vibrations when working with rock drills or impact wrenches was estimated to be 45 and 15 min a day, respectively, based on questionnaire information. These estimates were supported by field observations and discussions with the workers.

Table 4 shows the tremor measurements of workers who were exposed to the main tools and of the referents. The exposed workers had postural tremor with a higher frequency than the referents: 7.9 versus 7.2 Hz and 8.1 versus 7.0 Hz for the dominant and non-dominant hands, respectively. The exposed workers also had a tendency toward higher Tremor Intensity compared with the referents. This was statistically significant for postural tremor in the non-dominant hand and for rest tremor in the dominant hand.

**Table 4:** Hand tremor parameters for all subjects by exposure group

	Exposed (*N* = 55)			Referents (*N* = 48)			
	Arithmetic mean	SD	Min–max	Arithmetic mean	SD	Min–max	*p*
*Postural tremor, dominant hand*							
Tremor Intensity (ms^−2^)	0.16	0.09	0.06–0.66	0.14	0.06	0.06–0.34	
Log_10_ Tremor Intensity (ms^−2^)	−0.85	0.19	–	−0.90	0.17	–	0.12
Center Frequency (Hz)	7.9	1.2	5.5–11.1	7.2	1.6	1.3–10.6	0.02
Frequency Dispersion (Hz)	3.2	0.73	1.0–4.5	3.1	0.91	0.2–4.7	0.69
Harmonic Index	0.90	0.04	0.80–0.98	0.91	0.04	0.84–0.97	0.10
*Postural tremor, non*-*dominant hand* ^a^							
Tremor Intensity (ms^−2^)	0.17	0.11	0.06–0.63	0.13	0.04	0.07–0.26	
Log_10_ Tremor Intensity (ms^−2^)	−0.84	0.22	–	−0.93	0.14	–	0.03
Center Frequency (Hz)	8.1	1.3	5.2–10.6	7.0	1.3	4.7–9.9	<0.001
Frequency Dispersion (Hz)	3.6	0.6	1.6–4.7	3.3	0.9	0.7–5.0	0.16
Harmonic Index	0.88	0.05	0.76–0.96	0.89	0.04	0.79–0.98	0.19
*Rest tremor, dominant hand*							
Tremor Intensity (ms^−2^)	0.10	0.07	0.02–0.26	0.07	0.05	0.02–0.25	0.02
Center Frequency (Hz)	9.3	1.3	4.5–11.3	9.3	1.0	7.8–11.4	0.89
Frequency Dispersion (Hz)	2.7	0.9	0.8–4.9	3.0	1.0	0.2–5.4	0.11
Harmonic Index	0.91	0.03	0.82–0.98	0.91	0.03	0.85–0.99	0.76
*Rest tremor, non*-*dominant hand* ^a^							
Tremor Intensity (ms^−2^)	0.10	0.06	0.02–0.35	0.08	0.04	0.02–0.24	0.12
Center Frequency (Hz)	9.5	1.1	7.0–12.0	9.3	1.7	1.8–11.8	0.59
Frequency Dispersion (Hz)	2.8	0.8	0.7–5.6	3.0	1.1	0.3–5.6	0.15
Harmonic Index	0.91	0.03	0.84–0.97	0.90	0.04	0.82–0.98	0.35

^a^ Non-dominant hand: *N* = 102

**Fig. 1:**
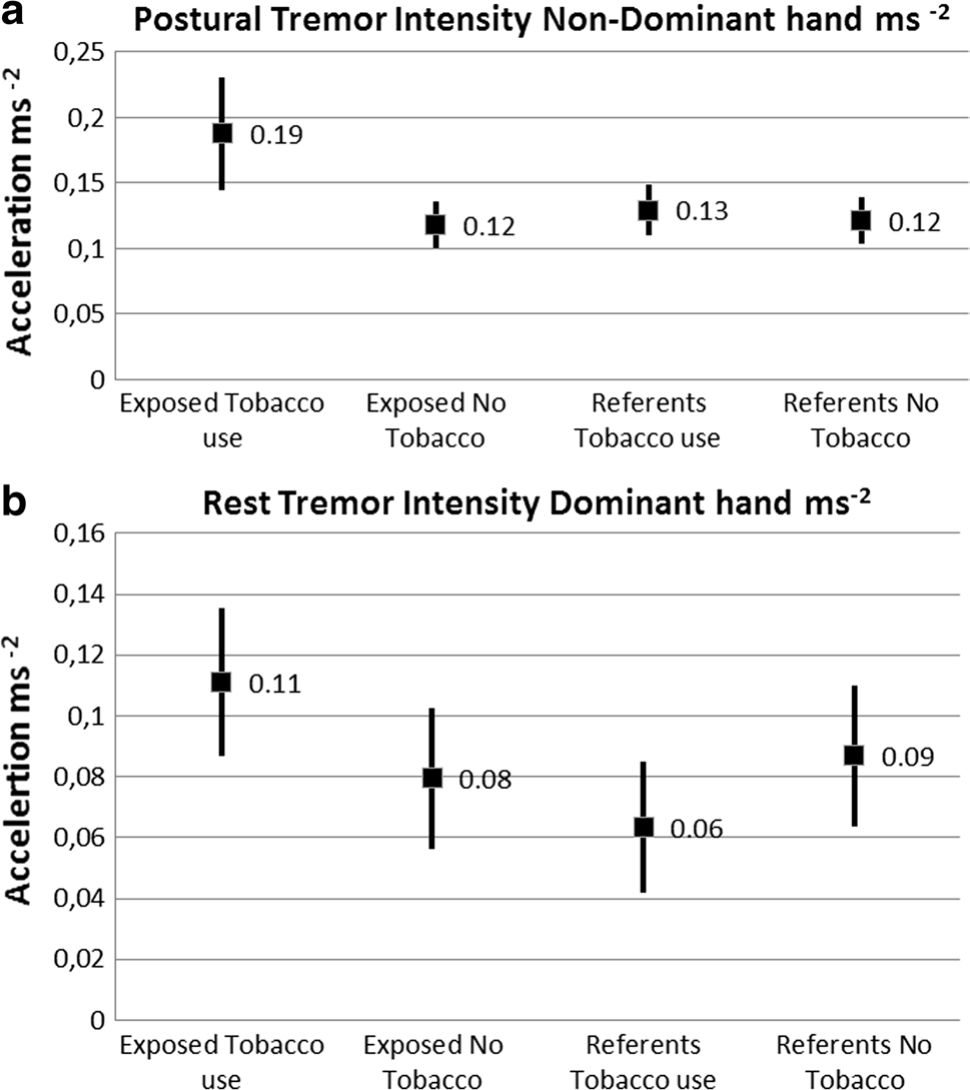
**a** and **b** The Tremor Intensity ms^−2^ (mean ± 95% CI) for postural tremor in the non-dominant hand and for rest tremor in the dominant hand, according to exposure status and serum cotinine levels (µg L^−1^) >5 as a marker of tobacco consumption. Mean cotinine level 632 (µg L^−1^) for the tobacco using exposed workers. Mean cotinine level 365 (µg L^−1^) for the tobacco using referents

Regression analyses showed strong associations between tobacco consumption, cumulative exposure and tremor parameters. Consequently, the subjects were stratified according to exposure status and smokers/users of smokeless tobacco for further assessment of interaction effects between exposure to vibration and tobacco consumption and tremor parameters. The unadjusted tremor values for some tremor parameters according to exposure status and tobacco consumption are illustrated in Fig. 1.

The results from further analysis of the combined effects from nicotine use and cumulative exposure adjusted for age are shown in Table 3. Among the tobacco users, increased Tremor Intensity was associated with higher cumulative exposure for postural as well as rest tremor in both hands. The Center Frequency of the postural tremor was also positively associated with the cumulative exposure in both hands. Among the nonusers of tobacco, no significant associations between cumulative exposure and tremor parameters were found. The participant in the reference group with HbA1c exceeding 6% did not have increased tremor, and his test results did not influence the associations between tremor parameters and cumulative exposure.

**Fig. 2:**
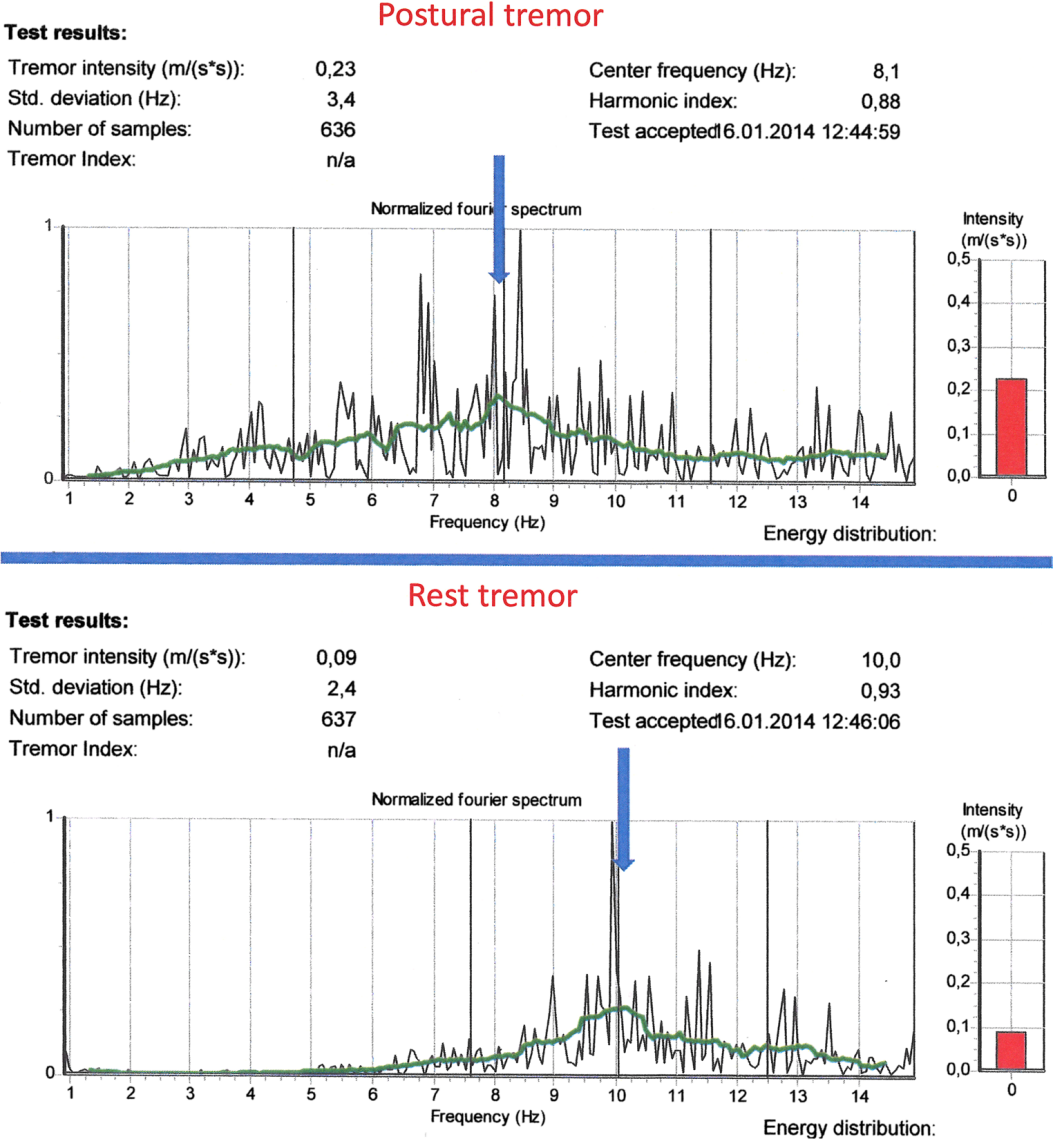
Illustration of tremor power spectra from the DPD TREMOR. Postural tremor versus rest tremor. Dominant Hand, same subject from the Rock Drill group. Rest tremor: Higher Center Frequency and narrower Frequency Dispersion than postural tremor. The Tremor Intensity is illustrated at the right

Table 5 shows the comparison between postural tremor and rest tremor parameters. The magnitude of the tremor, the Tremor Intensity, was lower when the hand was placed in a rest position. The Center Frequency increased significantly, from 7.5 to 9.3 Hz and from 7.6 to 9.4 Hz for the dominant and non-dominant hands, respectively, in the rest position. The tremor had a smaller Frequency Dispersion in the rest position, indicating that the power was concentrated at a narrower range of frequencies. Figure 2 gives an illustration of a typical power spectrum of one subject from the rock stabilization crew, illustrating that the tremor specter is shifted to a higher frequency.

**Table 5:** Comparison between characteristics of postural tremor and rest tremor for all subjects

	Postural tremor (*N* = 103) Arithmetic mean SD		Rest tremor (*N* = 103) Arithmetic mean SD		*p*
*Dominant hand*					
Tremor Intensity (ms^−2^)	0.15	0.08	0.09	0.06	<0.001
Center Frequency (Hz)	7.6	1.5	9.3	1.2	<0.001
Frequency Dispersion (Hz)	3.1	0.8	2.9	1.0	0.02
Harmonic Index	0.90	0.04	0.91	0.03	0.35
*Non*-*dominant hand* ^a^					
Tremor Intensity (ms^−2^)	0.15	0.09	0.09	0.06	<0.001
Center Frequency (Hz)	7.6	1.4	9.4	1.4	<0.001
Frequency Dispersion (Hz)	3.5	0.8	2.9	1.0	<0.001
Harmonic Index	0.88	0.05	0.90	0.03	<0.001

^a^ *N* = 102 for non-dominant hand

Nine of the subjects exposed to the main tools, eight from the rock stabilization crew and one from the guard rail crew were diagnosed with HAVS. In addition, three subjects unexposed to the main tools but with some exposure to other hand-held vibrating tools, had a clinical picture resembling a HAVS status. Among the nine subjects with the HAVS diagnosis, there were two who had only vascular abnormalities, four who had neurological disturbances, while three had both. Among the five subjects with vascular HAVS, two had stage 1, one had stage 2 and two had stage 3. The seven subjects with neurological HAVS were divided into one subject with stage 1, four subjects with stage 2 and two subjects with stage 3. Among the three subjects with both vascular and neurological HAVS, two had neurological HAVS stage 2, while one subject had stage 3 (not tabulated).

The subjects with HAVS had higher cumulative exposure, but they had also higher concentrations of serum cotinine and nicotine than the subjects who were free of HAVS. The magnitude of their postural tremor, the Tremor Intensity, was statistically significantly different between groups. The HAVS group had on average a postural Tremor Intensity of 0.24 ms^−2^ (dominant hand) and 0.23 (non-dominant hand) compared with 0.14 ms^−2^ for the subjects without this diagnosis (dominant hand) and 0.15 or 0.13 for exposed subjects versus referents free of HAVS (non-dominant hand). The HAVS group had also a postural tremor with a statistically significant higher frequency than the referents (Table 6).

**Table 6:** Background data, cumulative exposure measures and postural and rest Tremor Intensity and tremor Center Frequency among nine workers diagnosed with HAVS, exposed workers without HAVS and referents^a^

	Diagnosed with HAVS		Exposed no HAVS		Referents no HAVS		F	*p*
	Mean	SD	Mean	SD	Mean	SD		
	*N* = 9		*N* = 46^b^		*N* = 45			
Age	38.2	11.9	41.6	10.4	38.3	14.4	0.8	0.44
Cumulative exposure (ms^−2^ x h)	17,860	30,180	6470	16,070	0	0	–	
Log _10_ cumulative exposure (ms^−2^ x h)	3.60	0.92	3.25	0.74	0	0	418.1	<0.001^c,d^
Prevalence smoker/user smokeless tobacco “snus” (%)	78	–	63	–	47	–	2.1	0.12
sNicotine (µg L^−1^)	31	32	18	29	9	19	3.3	0.04^b^
sCotinine (µg L^−1**)**^	633	447	382	440	193	292	5.9	0.004^c,d^
sCDT (%)	0.7	0.3	0.7	0.2	0.7	0.2	–	
Log_10_ sCDT	−0.18	0.12	−0.17	0.11	−0.20	0.12	0.8	0.45
HbA1c (%)	5.5	0.2	5.3	0.2	5.2	0.4	2.5	0.09
*Postural tremor dominant hand*								
Tremor Intensity (ms^−2^)	0.24	0.17	0.14	0.06	0.14	0.06	–	
Log _10_ Tremor Intensity (ms^−2^)	−0.71	0.26	−0.88	0.17	−0.90	0.18	4.3	0.02^b,c^
Center Frequency (Hz)	8.4	1.4	7.7	1.2	7.2	1.7	3.4	0.04^c^
*Postural tremor non*-*dominant hand*								
Tremor Intensity(ms^−2^)	0.23	0.16	0.15	0.09	0.13	0.04	–	
Log _10_ Tremor Intensity (ms^−2^)	−0.72	0.25	−0.87	0.20	−0.92	0.14	4.7	0.01^b,c^
Center Frequency (Hz)	8.1	1.1	8.0	1.3	7.1	1.3	7.2	0.001^c,d^
*Rest tremor dominant hand*								
Tremor Intensity (ms^−2^)	0.12	0.08	0.10	0.06	0.07	0.05	3.0	0.055^c^
Center Frequency (Hz)	9.0	1.4	9.4	1.3	9.3	1.1	0.3	0.73
*Rest tremor non*-*dominant hand*								
Tremor Intensity (ms^−2^)	0.12	0.07	0.10	0.06	0.08	0.04	1.8	0.16
Center Frequency (Hz)	10.1	1.0	9.4	1.1	9.3	1.7	1.2	0.30

^a^ *N* = 100; three subjects with a clinical picture similar to HAVS were not included in analysis

^b^ *p* < 0.05 between the subjects with HAVS diagnosis and exposed subjects with no HAVS

^c^ *p* < 0.05 between the subjects with HAVS diagnosis and referents

^d^ *p* < 0.05 between the exposed subjects with no HAVS diagnosis and referents

Table 7 shows the association between a diagnosis of HAVS, cumulative exposure and the potential confounders age and sCotinine. After adjusting for these covariates, cumulative exposure was significantly (*p* = 0.03) associated with a diagnosis of HAVS.

**Table 7:** Odds ratio (OR) for being diagnosed with HAVS with increasing cumulative exposure to hand-arm vibration, adjusted for age and sCotinine

	OR	OR 95% C.I.		*p*
		Lower bound	Upper bound	
Cumulative exposure Log_10_ (ms^−2^ r.m.s. x h)	2.73	1.13	6.60	0.03
sCotinine (µg L^−1**)**^	1.002	1.00	1.004	0.07
Age <35 years	Ref = 1	Ref = 1	Ref = 1	
Age 35–44 years	0.21	0.02	2.21	0.19
Age 45–54 years	0.13	0.01	1.61	0.11
Age 55–64 years	0.32	0.03	3.52	0.35
Constant	0.006			0.002

The levels of serum cotinine did not act as a confounder affecting the association between exposure and the outcome of a HAVS diagnosis.

## Discussion

In this cross-sectional study of road maintenance workers, cumulative exposure was associated with increased postural and rest tremor and with postural tremor with higher frequency among smokers and users of smokeless tobacco. No statistically significant association was found between cumulative exposure and tremor parameters among nonusers of tobacco products. Rest tremor had a higher Center Frequency. Postural tremor parameters were more strongly associated with exposure than rest tremor.

The finding of increased postural tremor among the HAVS subjects indicated that tremor might be a part of the clinical picture of a HAVS diagnosis.

### Exposure assessment

Cumulative exposure was calculated from acceleration levels of the tools. The levels used were as given by Griffin et al. (2006), i.e., 17 ms^−2^ r.m.s. as the typical level for rock drills and 7 ms^−2^ r.m.s. for impact wrenches. These levels were confirmed using field measurements of vibration magnitude and questionnaire-based assessment of time used to operate the main handheld tools. Cumulative exposure was used as the exposure measure. The validation of vibration exposure levels using field measurements is considered a strength of this study.

The reliability of exposure–response relationships in studies of vibration-exposed workers has been questioned (Burström et al. 1998) in that studies have shown both over- and underestimation of the predicted risk (Burström et al. 2006). One study reported a large discrepancy in reported exposure time when comparing the answers on a questionnaire with a structured interview that included questions regarding estimated hand-held vibration exposure (Gerhardsson et al. 2005).

### Tremor measures related to hand-arm vibration exposure and to biomarkers

The CATSYS test system has been used in a number of studies of nervous system effects of exposure to neurotoxins but in only one published study of tremor in hand-arm vibration-exposed subjects (Edlund et al. 2015). In the present study, the testing time was set to 16.4, 2 s to stabilize and 14.4 s for recording, which is equal to the recording time applied in a number of studies (Edlund et al. 2015; Ellingsen et al. 2014; Wastensson et al. 2012).

Cumulative exposure to hand-arm vibration was statistically significantly and positively associated with increased postural and rest tremor among smokers and users of smokeless tobacco. The Center Frequency of the postural tremor, but not the rest tremor, was also significantly associated with cumulative exposure and with consumption of tobacco products (Table 3). These finding are in accordance with a study by Edlund et al. (2015) in which nicotine use was associated with increased postural Tremor Intensity and with higher tremor frequency, although the latter was found only for the dominant hand. Studies of manganese-exposed workers have shown increased tremor associated with the self-reported consumption of tobacco products (Bast-Pettersen et al. 2004, 2005) and with self-reported tobacco consumption as well as with cotinine levels in serum and urine (Ellingsen et al. 2006). Whether the observed associations between tobacco consumption and tremor parameters are related to a nicotine effect or to withdrawal from nicotine cannot be decided in the present study. However, we can document an association between tobacco consumption and tremor parameters.

Cotinine has a half-life of approximately 16 h compared with nicotine’s 2 h half-life, and therefore, cotinine values are better estimates of present nicotine use in the final hours prior to the serum sampling (Davis et al. 2015; Hukkanen et al. 2005). To our knowledge, this is the first study of tremor in vibration-exposed workers in which consumption of alcohol, nicotine and caffeine are assessed using biomarkers of exposure rather than by self-report. Edlund et al. (2015) used an interview to assess smoking habits and divided the subjects into nicotine users and nonusers. Self-report of alcohol and tobacco consumption has a number of weaknesses compared with biomarkers of this consumption. Gorber et al. (2009) found that self-report underestimated the true smoking prevalence in most studies included in a systematic review.

Because none of the participants in the present study had sCDT values exceeding the upper reference limit of the laboratory (>1.7%), there was no indication of excessive alcohol consumption at a level of daily consumption of 60–80 g of ethanol or more in any of the participants (Bortolotti et al. 2006). Thus, an influence of heavy drinking on the results is not suspected in the present study.

### Comparison between postural tremor and rest tremor

The results confirm that activation conditions have a large impact on tremor parameters. When comparing postural and rest tremor parameters, we found, in addition to the expected lower rest Tremor Intensity, a higher Center Frequency for the rest tremor. Further, the rest tremor had a narrower Frequency Dispersion, indicating that the power was concentrated at a narrower range of frequencies (Table 5). It has been suggested that rest tremor represents “a central tremor” (Deuschl et al. 2001) that to a larger extent is dominated by the 8–12 Hz oscillator. It is also possible that the recorded tremor is more affected by a component of finger tremor than hand tremor in the resting position, and finger tremor has been reported to have a higher frequency than hand tremor (Deuschl et al. 2001).

Because rest tremor has been proposed to represent a more “central tremor” (Deuschl et al. 2001), our findings of a strong association between cumulative exposure and increased postural tremor with a higher frequency and a weaker association between rest tremor and cumulative exposure dose could indicate that the increased tremor in the exposed group represents a “peripheral tremor.” Further, the lack of associations between tobacco consumption, cumulative exposure and rest tremor Center Frequency could indicate that the increased postural tremor Center Frequency among the smokers and users of smokeless tobacco was also due to a “peripheral tremor” effect from nicotine consumption among hand-arm vibration-exposed subjects.

### Tremor parameters related to HAVS diagnosis

The nine subjects exposed to the main tools who were diagnosed with HAVS had a Tremor Intensity of 0.24 ms^−2^ (dominant hand) and 0.23 (non-dominant hand), while the subjects without HAVS had a Tremor Intensity of 0.13–0.15, a difference that was statistically significant (Table 6). Edlund et al. (2015) found tremor intensities of 0.14 (dominant hand) and 0.12 (non-dominant hand) among 139 workers exposed to HAV, but these workers had not been diagnosed with HAVS. When comparing the Tremor Intensity of the subjects diagnosed with HAVS with subjects from other studies of working populations, the magnitude of the tremor was much higher than in subjects exposed to neurotoxins such as mercury (Bast-Pettersen et al. 2005; Wastensson et al. 2006) or manganese (Bast-Pettersen et al. 2004; Blond and Netterstrøm 2007; Ellingsen et al. 2008, 2015; Wastensson et al. 2012). In the present study, the subjects without HAVS had a Tremor Intensity similar to the values in the above-mentioned studies. Thus, a Tremor Intensity of 0.23–0.24 among the HAVS subjects is of clinical interest. The findings suggest that hand tremor may be a part of the clinical picture of HAVS, at least for hand-arm vibration-exposed subjects who are also tobacco users.

### HAVS diagnosis related to exposure, age and biomarkers

The subjects who were diagnosed with HAVS had a higher cumulative exposure to handheld vibrating tools than did the subjects without HAVS. However, there was considerable variation in cumulative exposure (Table 6). The concentrations of CDT found in the present study did not indicate heavy alcohol consumption in any subject, and this factor therefore probably did not influence the likelihood of a diagnosis of HAVS in the present study.

The vibration-exposed workers’ concentrations of cotinine and nicotine in serum were considerably higher than among the unexposed, indicating a higher consumption of tobacco products. One explanation for this could be that nicotine acts as a vasoconstrictor on small blood vessels (Powell 1998). A HAVS diagnosis, which to a certain degree is based on a clinical examination with a color chart and finger mapping (of vibration-induced white fingers), could therefore be affected by the subjects’ consumption of tobacco.

The likelihood of being diagnosed with HAVS was statistically significantly associated with cumulative exposure (Table 7). Higher cotinine concentrations as a biomarker of smoking and use of smokeless tobacco, although nonsignificantly associated with the likelihood of a HAVS diagnosis, were not confounding the associations between cumulative exposure and the likelihood of being diagnosed with HAVS.

### Aspects of validity, strengths and limitations

The high participation rate (97%) indicates that the findings in the present study are representative for male road maintenance workers with similar work tasks and exposure duration.

Other strengths of the study were that tremor was measured with a standardized tremor test and that potential confounders such as alcohol consumption, use of nicotine products and consumption of caffeine-containing drinks were assessed using biomarkers rather than assessment by self-report.

Because all of the participants were men, there were no potential gender effects on the tremor results, such as differences in tolerance to nicotine or alcohol that could confound associations between exposure and outcome. However, caution is warranted if these findings are to be generalized to female subjects.

Some cautions must be made when interpreting the exposure parameters. We used the same main tool vibration level for all subjects who used the respective tools. It is, however, possible that individual factors such as working technique and grip force has an influence on the vibration exposure on the individual level. Such differences could impose a misclassification of exposure. It has also been debated whether the frequency weighting according to ISO 5349-2 that was used in the present study is the most appropriate measure of exposure to hand-arm vibration (Brammer and Pitts 2012).

We did not have measurement data to assess exposure to hand-held vibrating tools other than rock drills and impact wrenches. Instead, such exposure was introduced into models by indicator variables. These did not show any associations with the outcome. The fact that indicators that represent such exposures in the regression models were not associated with tremor parameters may be a spurious finding, resulting from exposure misclassification that is known to dilute a true association, if present (Rothman et al. 2008).

As with all cross-sectional studies, inference should be made with caution when drawing conclusions about associations between exposure and possible effects. For instance, we cannot rule out the possibility that that there has been a selection out from high-exposed jobs of workers who have experienced untoward effects from exposure to hand-held vibrating tools. Such selection, equal to a healthy worker effect, due to exclusion from exposure, may dilute the true associations between exposure and outcome. Future research using a longitudinal design with better control over such selection phenomena may validate the findings of the present study.

Despite the limitations that a cross-sectional design entails, our findings indicate that exposure to vibrating hand tools was related to the differences in hand tremor between the exposed and the referents.

## Conclusions

The main findings in this cross-sectional study of road maintenance workers indicate an association between cumulative exposure to hand-held vibrating tools, tremor parameters and consumption of tobacco products. Cumulative exposure was associated with increased postural and rest tremor and postural tremor with higher tremor frequency among smokers and users of smokeless tobacco. The hand position (activation condition) is important when testing for tremor. Rest tremor had a higher Center Frequency. Postural tremor parameters were more strongly associated with exposure than rest tremor.

The finding of increased postural tremor among the HAVS subjects indicated that tremor might be a part of the clinical picture of a HAVS diagnosis.

The findings of increased tremor among subjects exposed to hand-arm vibration indicate that preventive actions to reduce exposure are warranted among workers who are exposed at their present levels. As with all cross-sectional studies, causal inferences should be made with caution when drawing conclusions about associations between exposure and possible effects. Future research using longitudinal design is required to validate the findings of the present study.
